# Influential Factors Associated with Consecutive Crash Severity: A Two-Level Logistic Modeling Approach

**DOI:** 10.3390/ijerph17155623

**Published:** 2020-08-04

**Authors:** Fanyu Meng, Pengpeng Xu, Cancan Song, Kun Gao, Zichu Zhou, Lili Yang

**Affiliations:** 1 Academy for Advanced Interdisciplinary Studies, Southern University of Science and Technology, Shenzhen 518000, China; mengfy@sustech.edu.cn; 2 Department of Statistics and Data Science, Southern University of Science and Technology, Shenzhen 518000, China; 3 Department of Civil Engineering, The University of Hong Kong, Hong Kong 999077, China; 4 Key Laboratory of Road and Traffic Engineering of the Ministry of Education, Tongji University, Shanghai 201804, China; songcancan@tongji.edu.cn (C.S.); 1410705@tongji.edu.cn (Z.Z.); 5 Department of Architecture and Civil Engineering, Chalmers University of Technology, 41296 Göteborg, Sweden; gkun@chalmers.se

**Keywords:** consecutive crash, crash severity, traffic hazard, multi-level model, unobserved heterogeneity

## Abstract

A consecutive crash series is composed by a primary crash and one or more subsequent secondary crashes that occur immediately within a certain distance. The crash mechanism of a consecutive crash series is distinctive, as it is different from common primary and secondary crashes mainly caused by queuing effects and chain-reaction crashes that involve multiple collisions in one crash. It commonly affects a large area of road space and possibly causes congestions and significant delays in evacuation and clearance. This study identified the influential factors determining the severity of primary and secondary crashes in a consecutive crash series. Basic, random-effects, random-parameters, and two-level binary logistic regression models were established based on crash data collected on the freeway network of Guizhou Province, China in 2018, of which 349 were identified as consecutive crashes. According to the model performance metrics, the two-level logistic model outperformed the other three models. On the crash level, double-vehicle primary crash had a negative association with the severity of secondary consecutive crashes, and the involvement of trucks in the secondary consecutive crash had a positive contribution to its crash severity. On a road segment level, speed limit, traffic volume, tunnel, and extreme weather conditions such as rainy and cloudy days had positive effects on consecutive crash severity, while the number of lanes was negatively associated with consecutive crash severity. Policy suggestions are made to alleviate the severity of consecutive crashes by reminding the drivers with real-time potential hazards of severe consecutive crashes and providing educative programs to specific groups of drivers.

## 1. Introduction

With the increasing number of traffic crashes occurring in the past decade, both researchers and practitioners in the road safety field have been focusing on topics such as crash risks, severity levels, and safety behaviors to propose prevention measures for alleviating social and economic losses [1,2,3,4,5,6,7]. Some scholars are focused on investigating the risk factors and severity levels of crashes in specific scenarios, as the mechanisms of crashes may vary considerably in different situations, e.g., with different traffic conditions, involving different numbers of vehicles, or caused by different at-fault driver groups. Secondary crashes, defined as the crashes occurring spatially and temporally within the impact area of a preceding crash, have always been considered a special crash scenario. They commonly occur as a consequence of the primary crash and may possibly cause greater burdens to the traffic system, as multiple crashes may severely block the roads and causes congestion, hindering victim evacuation and road clearance activities.

The occurrence of secondary crashes has been the main focus of previous research on this topic. Some studies have explored the occurrence of such type of crashes and its contributory factors, most of which incorporated a logistic regression to model the probability of occurrence [8,9,10], and some have adopted variants of logistic regression for addressing specific modeling issues [11,12]. Specifically, Xu et al. [11] have proven the existence of unobserved heterogeneity by adopting a random-effects logit model to discover the contributions of various factors on secondary crash propensity. Common understanding has formed that the occurrence of secondary crashes is significantly dependent on the duration of the primary crash, queue length, traffic condition, and road geometric design [13]. Various identification methods for secondary crashes applied in previous studies, including static methods with fixed spatial and temporal thresholds (usually high in numbers), and dynamic methods such as shock wave and speed contour methods, are both based on the assumption that congestion is incurred by the primary crash given a certain period of time [9,13].

Conceptually, secondary crashes are induced by queuing effects caused by primary crashes, and thus the effective area of the primary crash can be relatively large in time and space. In previous research on secondary crashes with static identification methods, the distance between the primary and secondary crashes are usually longer than 2 miles and the time gaps are larger than 2 h [14,15]. With this assumption, the traffic hazards produced by the primary crash are cumulative in time and space and spread through the queuing downstream vehicles.

There is a special type of immediate secondary crash that occurs in a considerably short period of time within a vicinity of the primary crash hasn’t gained enough attention in previous research. When the secondary crash happens shortly after the primary crash, traffic queues haven’t formed and the clearance of primary crash doesn’t apply. In this case, congestion is no longer a factor that induces the subsequent secondary crash, and the traffic hazards produced by the primary crash transmit mainly through vehicular interactions at a near-free-flow speed, similar to the mechanism of a chain-reaction (multiple-vehicle) crash. In this paper, the aforementioned primary crash and the subsequent immediate secondary crash is named as a consecutive crash (CC) series. One primary crash is possible to induce one or more secondary CCs. By definition, a CC series is different from a chain-reaction crash in that the latter is considered as one single crash with multiple vehicles involved as a result, and can normally be decomposed into several collisions, but a CC series is a group of multiple crashes, each of which can involve single or multiple vehicles.

As many similarities between CCs and chain-reaction crashes exist, the crash mechanisms of the two can be analogized. Chain-reaction crashes have been proven to be caused by a sudden change of the lead vehicle, e.g., a sudden slowdown or lane switch [16,17]. Traffic condition and drivers’ reaction speed have been found to affect the occurrences of a chain-reaction crash based on simulation methods [18]. In addition to simple chain-reaction crashes, traffic hazards may also spread backwards in a non-linear or irregular manner based on traffic wave theory. Imagine that the drivers of the following vehicles have different alertness levels and reaction swiftness, the same hazard produced in the front may cause different driving behaviors and vehicular interactions in its behind. Hence, collisions may not always occur to adjacent vehicles in the same lane. Instead, secondary crashes may happen several vehicles away from the primary crash shortly within its influential area, and then subsequently induce a series of CCs in a short period of time. This effect is also probable to be magnified on high speed roads and with busy traffic, such as on freeways, resulting in higher CC risks and severity levels [16,18].

Although there are considerable similarities between CCs and chain-reaction crashes, their impacts on the traffic system and society are quite different. CCs can cause relatively more burdens to the affected road and traffic because they involve multiple crashes in a limited road area, which possibly causes congestion and are difficult to evacuate. Clearance and traffic management of multiple crashes within a limited area of a road network are also relatively more cumbersome, and hence injuries may not be promptly transferred to hospital and higher injury severity or even more fatalities may result. Despite these challenges, little research has been focused on understanding the crucial aspects of this type of crash. Some experience from previous studies on the occurrence of primary and secondary crashes can be referred to, but they hardly addressed crash severity of secondary crashes and its influential factors, not to mention the severity issues of CCs.

This study aims to identify the factors associated with a CC series (including a primary CC and at least one secondary CC) on freeways while addressing unobserved spatial heterogeneity in the dataset. The crash data used in this study include 8779 crashes on the freeway network of Guizhou Province, China in 2018, from which 349 crashes involved in CC series were identified based on a static identification method. Factors including road geometric design, traffic conditions, environmental and crash attributes of the primary and secondary CCs are incorporated into the model with elaborated interaction terms. A basic, a random-effects (RE), a random-parameters (RP) and a two-level binary logistic regression are estimated and compared to quantify the relationships between CC severity and various influential factors. The best model form for modeling CC severity while dealing with unobserved heterogeneity and data hierarchy is selected based on the AIC values and other commonly used metrics. Significant influential factors are identified based on the model form with the best performance and discussed accordingly. Policies are also suggested to alleviate crash severity levels of CC on freeways and mitigate the potential losses.

## 2. Methods

### 2.1. Data

The crash database used in this study was provided by Guizhou Traffic Information and Emergency Control Center, affiliated to the Department of Transportation of the Guizhou Province. The data were originally recorded by onsite traffic police officers for all crashes on national, provincial and local freeways in the province. Data on the speed limit and monthly traffic volume in 2018 was provided by the Department of Transportation of Guizhou Province. In this study, a static identification method was applied to identify CCs from the crash database based on two conditions: (1) a secondary crash occurs in the downstream of the primary crash, and (2) the secondary is within 1 km in space and 1 min in time from the primary crash [13]. In total, there were 135 primary crashes and 214 immediate secondary crashes extracted from the database, resulting in an average of 1.59 secondary CCs per primary CC. To facilitate the understanding of influential factors of CCs and the differences between the contributory factors to the severity of primary and secondary CCs, all of these 349 crashes were included in the modeling.

The Transport Department of Guizhou has divided the road networks into 79 segments for management purposes. The length of these segments ranges from 40–80 km. Apart from their different locations and geographical and environmental conditions, different segments may be also under surveillance and management of different police departments from different cities/towns. The 349 crashes extracted for this study covered 12 out of the 79 road segments.

The dependent variable was extracted from the crash records as the severity level of each relevant crash. Given that the fatal crashes only accounted for a very small proportion among both the total crashes and the 349 CCs incorporated in this study (less than 1%), the dependent variable was set to contain two categories: severe (with at least one injury caused) and non-severe (with property damages only). The dichotomous nature of the dependent variable gives rise to the choices on model forms of this study.

Environmental factors and road geometric design attributes for each crash were recorded directly in the crash database, including time, location, weather, etc. The traffic volume information of the road segment where the crash happens was extracted based on the location information of each crash and transformed into 10^6^ units as the numbers were usually very large. Given the location of each crash, the speed limit and number of lanes of the road segment was also collected. If a crash occurred in a tunnel or on a bridge, the information was also recorded by the variable “location”, as the special driving environments may affect the transmission of the hazards produced by the primary crashes.

Crash-related characteristics such as the number of vehicles involved, crash type, and various types of vehicle involvement were also extracted from the database. Rear-end, rollover, and side-swipe crashes are worthy of special attention as three categories of the variable, crash type, with hitting objects as the base level. A standardized vehicle type categorization scheme of China was adopted in the database, classifying all vehicles into two main categories: light vehicles such as passenger cars and light commercial vehicles, and heavy vehicles which include trucks, buses, and trailer trucks. Heavy vehicles deserve more attention in this study as these vehicles are large in size and likely to induce more and propagate further hazardous driving conditions to vehicles in behind [11,19]. The involvements of truck, trailer truck, and bus in each crash were formulated into tree respective dummy variables to represent whether these three types of massive vehicles were involved. Number of vehicles involved in a crash is presumably contributive to the severity level of a crash on freeways and was also adopted as a dummy variable with the multiple-vehicle crashes as the baseline level. Besides, primary and secondary crashes were differentiated by the variable, “secondary CC”, as the contributary factors to the severity levels of primary and secondary CCs are assumed to be different. Interaction effects of this variable with other environmental factors and crash attributes are readily adopted in the models, to test the contributions of these factors on the severity levels of CCs.

As secondary CCs are by nature a result of the hazards produced by the corresponding primary crash, the crash severity of a secondary CC is assumed to be affected by the crash-related attributes of the primary crash. Hence, attributes of the primary crashes including crash type, number of vehicles involved, and involvements of various types of heavy vehicles (i.e., truck, trailer truck and bus) were delineated for all observations. The interaction terms between crash attributes of the primary crash and “secondary CC” were also introduced to measure the effects of crash-related attributes of the primary crash on the severity level(s) of its related secondary CC(s).

**Figure 1:**
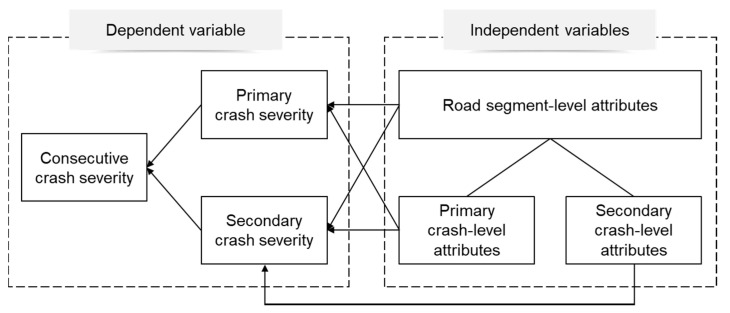
Conceptualization of the model structure for the severity of CC.

The descriptive statistics of the dependent and independent variables are shown in Table 1. For continuous variables, the sample means and standard deviations were provided. Categorical variables were all coded into dummy variables, and the percentage of each category together with that of the base level were provided. In addition, the conceptualization of the hierarchical data structure has been displayed in Figure 1, illustrating the two-level settings and the rationale for incorporating interaction terms.

### 2.2. Binary Logistic Regression

Logistic regression has been proven to be effective and widely used in measuring the relationships between binary injury outcomes, as the link function can transfer a linear function into a continuous probability function ranging from 0 to 1 [1,2,3,20,21]. Let $Y_{i}$ denote the outcome of CC severity i. $Y_{i}=1$ means that crash i is a severe crash, while $Y_{i}=0$ means that crash i is a non-severe crash. A binary logistic function is used to link the probability of $Y_{i}=1$ (denoted as $\pi_{i}$) with the independent variables as follows [22]:

(1) $$\mathrm{logit}(\pi_{i})=\log(\frac{\pi_{i}}{1-\pi_{i}})=\beta_{0}+\sum_{k=1}^{K}\beta_{k}X_{ik}+\varepsilon_{i}$$

where $X_{ik}$ is the value of the kth independent variable for crash i, $\beta_{0}$ is the intercept of the model, $\beta_{k}$ is the estimated coefficient for $X_{ik}$, and $\varepsilon_{i}$ is the random error term following a logistic distribution.

### 2.3. Random-Effects Logistic Model

Unobserved heterogeneity has been shown to widely exist in road crash modeling [20,21,23,24,25,26,27], as factors that were not accounted for by the independent variables may induce individual heterogenous effects on the outcome variable [26]. To address the unobserved heterogeneity possibly existing in our dataset, a RE modeling approach was adopted by incorporating a random intercept term, $v_{i}$, which was normally distributed across individual observations with a mean of 0 and a standard deviation of $\sigma_{v}$. The RE logistic function is formulated as follows:

(2) $$\mathrm{logit}(\pi_{i})=\log(\frac{\pi_{i}}{1-\pi_{i}})=\beta_{0}+\sum_{k=1}^{K}\beta_{k}X_{ik}+v_{i}+\varepsilon_{i}$$

A maximum likelihood estimation method is applied to estimate the coefficients, $\beta_{0}$ and $\beta_{k}$ [22]. In addition to the coefficients for the independent variables, the variance of the random intercept, $\sigma_{v}$ is also estimated simultaneously. Statistically, scholars commonly use another parameter, ρ, to represent the proportion of variance explained by the random effect [28]:

(3) $$\rho =\frac{\sigma_{v}^{2}}{\sigma_{v}^{2}+\sigma_{\varepsilon }^{2}}$$

where $\sigma_{\varepsilon }^{2}$ is the variance of $\varepsilon_{i}$.

### 2.4. Random-Parameters Logistic Model

While RE model is considered as a special case of a RP model where only the constant term is assumed to be random [26], a RP model has become increasingly prevalent in recent traffic safety studies due to its ability to address the heterogeneity in all independent variables. A RP modeling approach was therefore used by allowing the estimated coefficients for desired independent variables to vary across observations, as a comparison with the RE model. The RP logistic function is described as function (1), with the estimated coefficient $\beta_{k}$ allowed to be random as follows:

(4) $$\beta_{k}={\bar{\beta }}_{k}+\mu_{ik}$$

where ${\bar{\beta }}_{k}$ is the estimated mean of the kth coefficient, and $\mu_{ik}$ is a random term following a normal distribution with mean of zero and variance of $\sigma_{k}^{2}$.

A simulated maximum likelihood estimation method with 200 Halton draws is applied to estimate the coefficients, ${\bar{\beta }}_{k}$ and $\sigma_{k}$ [22]. A *Z*-test was applied to each estimated coefficient, and only the coefficients with the a significant mean, ${\bar{\beta }}_{k}$, and a significant standard deviation (SD), $\sigma_{k}$, were treated as random coefficients.

### 2.5. Two-Level Logistic Model

Previous studies have found that as most crash data were hierarchical in nature, spatial heterogeneity may exist on the higher level(s) of the hierarchy [29,30,31]. Vanlaar [32] found in his research on drunk driving that ignoring a hierarchical data structure could lead to underestimation of standard errors in predictors. Dupont et al. [33] also pointed out that traditional model structures ignoring hierarchical heterogeneity in the data structure might induce problematic inferences and conclusions. To address the within-road-segment correlation while capturing spatial heterogeneity across various road segments, a two-level modeling scheme was proposed here. In this case, level 1 was the crash level and level 2 was the road segment level. The basic logistic function should be rewritten into a two-level model form as follows:

(5) $$\mathrm{logit}(\pi_{ij})=\log(\frac{\pi_{ij}}{1-\pi_{ij}})=\beta_{0j}+\sum_{k=1}^{K}\beta_{1jk}X_{ijk}+\varepsilon_{ij}$$

where j is the index of road segment, $\beta_{0j}$ is the crash-level intercept and $\beta_{1jk}$ is the estimated coefficient of covariate $X_{ijk}$. Note that $\beta_{0j}$ and $\beta_{1jk}$ both vary across road segments (i.e., on level 2) and $\varepsilon_{ij}$ varies across all observations (i.e., on level 1). To address the cross-road-segment variations, the level 2 model is specified as follows:

(6) $$\begin{array}{l} \beta_{0j}=\gamma_{00}+\sum_{l=1}^{L}\gamma_{0l}Z_{jl}+\mu_{0j}\\ \beta_{1jk}=\gamma_{1k}+\mu_{1jk} \end{array}$$

where $\gamma_{00}$ and $\gamma_{1k}$ are the fixed intercepts on the road segment level; $Z_{jl}$ is the lth road-segment-level independent variable for segment j; $\gamma_{0l}$ is the estimated fixed coefficient for $Z_{jl}$; $\mu_{0j}$ and $\mu_{1jk}$ are the random effects varying across road segments for the crash-level intercept and crash-level covariate k with means of zero and variances of $\sigma_{0}^{2}$ and $\sigma_{k}^{2}$, respectively [34]. A similar simulated maximum likelihood estimation method with 200 Halton draws was used to accomplish model estimations. A *t*-test was performed for all estimated parameters including the random and fixed parameters on both crash and road segment levels.

### 2.6. Elasticity Analysis

As the estimated coefficients may not always directly represents the effects that a contributory factor has on the indicator, an elasticity analysis is necessary for quantifying the effect of each independent variable based on the observed and estimated information [28,35,36]. The elasticity for a continuous independent variable k on the probability of a severe crash is calculated from the partial derivative of each observations [28]:

(7) $$E_{ik}=\frac{\partial \pi_{i}}{\partial X_{ik}}\frac{X_{ik}}{\pi_{i}}$$

where the $E_{ik}$ is the elasticity outcome for continuous variable k of crash observation i. As the probability for a crash to be severe is not differentiable with dummy independent variables, a pseudo-elasticity is defined for indicators as follows [33,34]:

(8) $$E_{ik}^{\left(p\right)}=\frac{\partial \pi_{i}(X_{ik}=1)-\partial \pi_{i}(X_{ik}=0)}{\partial \pi_{i}(X_{ik}=0)}$$

where $E_{ik}^{\left(p\right)}$ is the pseudo elasticity of dummy variable k of crash observation i. The final elasticity of a variable is calculated as the sample mean of the elasticity outcomes for all observations.

### 2.7. Model Comparison

To facilitate model comparison, likelihood-based model performance indices such as log-likelihood and the Akaike Information Criterion (AIC) value were calculated. For the RE, RP, and two-level logistic regressions, a McFadden Pseudo $R^{2}$ value was also calculated as it is widely used as a measure for model fitting evaluation for simulated-based model estimations [22]. Similar to $R^{2}$ value in ordinary least square estimation, a McFadden Pseudo $R^{2}$ value varies between 0 to 1, and a value closer to 1 indicates a better model fit.

Moreover, the AIC is capable of reflecting the amount of information loss in each model [37] given the likelihood performance and number of predictors used in the model. The definition of the AIC value is as follows:

(9) $$AIC=2n-2\ln(\hat{L})$$

where n is the number of estimated parameters, and $\hat{L}$ is the maximum value of the likelihood function for the model. The model with the lowest AIC value has the best performance in terms of using as few parameters as possible to maximize the likelihood function of the model.

## 3. Results

To identify the significant factors associated with the severity level of CC series and compare the differences in influential factors between primary and secondary CCs, four models based on the logit link function have been estimated: basic, RE, RP, and two-level logistic models. The same set of dependent and independent variables were incorporated into all four proposed models. The environmental (level 2) independent variables and the crash-related (level 1) independent variables were all included. To test the contributions of attributes of the primary crash on the crash severity of its corresponding CC(s), the interaction terms between the crash-related attributes of the primary crash and the indicator variable representing secondary CC (namely “consecutive secondary crash” in Table 1) were adopted in the models. Besides, the indicator variable for secondary CC was also interacted with environmental variables and incorporated in all four models, to test the direct effects of the environmental variables on CC severity. Spearman’s correlation tests were performed for all pairs of independent variables to identify possible strong collinearities from the variable set [28]. All Spearman’s correlation values were smaller than 0.5, indicating that no strong collinearity was observed from the variables.

For the basic and RE binary logistic models, all independent variables in the aforementioned variable set were first adopted with their estimated parameters tested for statistical significance. In the final results in Table 2, the insignificant continuous variables and the categorical variables with all sub-categories that were insignificant at the 90% confidence level have been excluded. In the basic logistic model, ten coefficients were significant at the 95% confidence level and two coefficients (for truck involvement and tunnel × secondary CC) were marginally significant at the 90% confidence level. In the RE model, the same significant variables were found, but the random effect turned out to be insignificant, reflecting that the random intercept was not adequate to address the unobserved heterogeneity in the dataset.

For the RP model, a stepwise method for selecting proper RPs was adopted. The variables were set to be random one by one following an order from the lower-level variable (crash level) to the higher-level (environmental) variable, and then to the interaction terms (the order for adopting random interaction terms was also from the lower to the higher level). Note that parameters of all categories for a categorical variable were considered to be random in the same step. Only the variables with both the mean and the standard deviation (S.D.) were significant at the 95% confidence level were kept random, and the rest were all set as fixed parameters. In the final model (see Table 2), only the significant continuous variables and the categorical variables with at least one significant category were kept (confidence level is set to be 90%). Finally, nine fixed coefficients were significant at the 95% confidence level or above and two were marginally significant at the 90% confidence level. One variable was found to have a significant mean and a significant SD at the same time.

According to the model forms of the two-level binary logistic model, a random term for each crash-level variable was estimated. As the interaction terms between the secondary CC indicator and crash-level attributes also represented crash-level effects, random effects were allowed for these interaction terms as well. For random term selection, only the variables with either a significant fixed effect or a significant random effect was adopted in the final model.

That is to say, the crash-level variables (including the aforementioned interactions) whose fixed and random terms were both insignificant at the 95% confidence level were excluded as no significant effect were found for this variable from both levels. For level-2 variables, statistically insignificant continuous variables and categorical variables whose all sub-categories were insignificant have been excluded in the final results. Six level-2 variables had a coefficient that was significant at the 95% level or above; two level-1 interaction terms were significant at the 95% level. Although the estimated fixed intercept was insignificant, the random effect of the intercept varying across road segments was significant at the 95% confidence level.

Major metrics reflecting model performances of the four proposed models for severity of CC series are listed and compared in Table 3. Based on the log-likelihood value at convergence and the AIC, the RE model (log-likelihood = −162.70, AIC = 355.4) didn’t improve in model performance compared to the basic binary logistic model (log-likelihood = −162.70, AIC = 353.4). The RP model had a relatively higher log-likelihood at convergence (−159.88) and lower AIC (349.8) than the basic and the RE logistic regression, indicating that the full heterogeneity model can better address the unobserved heterogeneity in the data and achieve a better model performance. The two-level logistic regression outperformed the other three models and had a substantial enhancement in all of the three major metrics, especially in the AIC value (325.4), meaning that within-segment correlation exists in our dataset and the two-level model structure fits the crash dataset better than the other three proposed model forms. Hence, the elasticity analyses were performed for the optimal model choice, the two-level model, to facilitate the discussion process. As shown in Table 4, all of the elasticity values were significant at the 99% confidence level, and the results echo with the estimated coefficients in Table 2. The elasticity values for five main effects were positive, namely speed limit, traffic volume, rainy, cloudy and tunnel. Two other main effects had a negative elasticity value: the number of lanes and the intercept.

## 4. Discussion

As the two-level binary logistic regression model outperformed the other three models in modeling the crash severity for CC series, the two-level form is considered superior among the tested model forms with the most unbiased results. Hence, the following discussions are mainly based on the estimation results of the two-level binary logistic model.

As shown in Table 2, the random terms for the crash-level variables, the interaction between truck involvement and secondary CC, and the interaction between double-vehicle primary crash and secondary CC were not significant, indicating that although these two factors have significant fixed contributions on secondary CC severity, the road-segment-level spatial heterogeneity doesn’t locate in these two variables. Instead, the SD of the random intercept of the road segment level was significant at the 95% confidence level, indicating that spatial heterogeneity exists on the road segment level and locates mainly in the intercept. Given that the two-level structure has been proven more robust and unbiased, the individually heterogenous effect of “cloudy” in the RP regression was rather a false alarm.

### 4.1. Crash-Related Factors and Interactions

The interaction between truck involvement and secondary CC crash was found to be positively associated with the propensity of a severe crash (coefficient = 2.80). This result indicates that if trucks were involved in a secondary CC, the severity outcome of the crash tended to be more severe compared to the one without trucks involved. Similar results have been found in previous research in which the involvement of truck was reported to significantly increase the severity of the crash under various circumstances, provided that trucks are massive in weight and size and potentially more disruptive in a traffic collision [7,38,39]. Zhou and Zhang [40] studied the potential hazardous driving behaviors of commercial truck drivers and identified that 40% of truck drivers tended to drive in a substantially dangerous way. Secondary CCs occur under the direct effect of the primary crash immediately. For secondary crashes in a CC series, taking a swift action to the traffic hazards generated from the primary crash is crucial to alleviating its crash severity, yet trucks are cumbersome and slow down the changes in movements to a large extend. The nature of massiveness, the hazardous driving behaviors, and the cumbersomeness in reaction of a truck are the possible reasons that more severe secondary CCs tend to be induced when trucks are involved.

The estimated coefficient for the interaction term between two-vehicle primary crash and secondary CC was significantly negative at the 95% confidence level (coefficient = −1.59). Compared to a primary crash with three or more vehicles involved, a primary crash involving two vehicles had a lower propensity to cause a severe CC in its behind. Nagatani and Yonekura [16], Sugiyama and Nagatani [17], and Nagatani [18] investigated the mechanism of multiple-vehicle crashes with various hazardous inputs such as a sudden lane change and a sudden brake based on a car-following model, similar to the mechanisms of CCs except for the less strong assumptions in the regularity of vehicular movements and reaction behaviors. Under most circumstances, the following vehicles’ sensitivity (agility in reaction) was associated with the occurrences of chain-reaction crashes and the number of vehicles involved in the crash chain. In the case of CCs, similar car-following behaviors are also valid and reaction speed is even more crucial if the primary crash involves more than three vehicles and engages a relatively larger area of the road and generates a larger number of traffic hazards from multiple origins, possibly resulting in multiple unsafe behaviors in the traffic behind. Compared with two-vehicle primary crashes, the more complicated combination of behaviors can possibly overload the following vehicles’ reaction capabilities and thus cause a more severe secondary CC subsequently.

Although multiple-vehicle primary crashes were proven to have a higher likelihood of severe secondary CCs than two-vehicle primary crashes, the number of vehicles involved in the primary crash was not positively associated with the severity of secondary CCs. The estimated coefficient for the interaction between single-vehicle primary crash and secondary CC was insignificant, meaning that no evidence could be found to differentiate the effect of a single-vehicle primary crash on the severity of the secondary CC from that of a multiple-vehicle primary crash as single-vehicle crashes usually occur under extreme circumstances, such as losing control of the vehicle or rollover crashes [41] and could have ambiguous effects on the severity of the secondary CCs.

### 4.2. Environmental Factors

In the two-level binary logistic model, six environmental factors were significant at the 95% level or above, five of which had a positive effect on the severities of crashes in a CC series based on the estimation results, including speed limit, traffic volume, rainy, cloudy, tunnel, and bridge. According to the elasticity analyses, the sample means of the marginal effects of these variables were also significantly positive. The number of lanes was the only environmental variable that was negatively associated with the crash severity of CC series.

The elasticity value of speed limit was 2.60, indicating that 1% increase in speed limit will lead to a 2.6% increase in the probability of a severe CC on average. This result reveals a similar effect of speed limit to positively contribute to the probability of severe crashes on freeways or highways [38,42,43], especially to those fatal crashes. As vehicles tend to have a higher speed driving on a freeway with a higher speed limit, drivers have a relatively shorter time for reaction to emergencies and take action to avoid a fierce collision. Hence, for both primary and secondary crashes in a CC series, a higher speed limit is more likely to result in severe crash outcomes.

Traffic volume of the road segment where the CC occurred had a significantly positive effect on CC severity (coefficient = 1.41) and a significantly positive elasticity effect (1.64). This result suggests that the denser the traffic is, the more likely for a CC to be severe. Zeng, et al. [44] studied the severity crashes on freeways and found that traffic volume significantly affected crash severity and impacted the threshold between median and severe crashes. Wang, et al. [45] also concluded that a higher traffic volume may increase the distance gap between the primary and the secondary crash based on a shock wave method. Although this result may apply to CCs, the longer distance gap could still add inequality in the spread pattern of the traffic hazards produced by the primary CC, especially within a time period that is short enough, and hence result in more severe secondary CCs.

The number of lanes was the only environmental factor that had a significantly negative effect on the severity of crashes in a CC series among all the factors estimated (coefficient = −4.29, *p* = 0.008). The elasticity value of this variable was −5.11 and significant at the 99% level, indicating that 1% increase in the number of lanes on one side of the freeway may decrease the probability of a CC to be severe by 5.77%. Compared with a CC occurring on a three-lane freeway, the same CC occurring on a two-lane freeway had approximately 1.9 times higher probability to be severe if other factors hold the same. In previous research, hazardous lane-keeping behaviors of the front vehicle has been widely proven to affect crash injury severity positively. Jamal, et al. [46] studied the vehicular crash severity in Saudi Arabia and identified that a sudden deviation from the lane of the at-fault vehicle caused a higher crash severity. Shao, et al. [47] applied a RP ordered logit model to prove that a sudden stop of the front vehicle in the same lane significantly raised the injury severity of rear-end crashes with trucks involved. Hence, the severity of the primary crash in a CC series can be affected by the hazardous lane-related behaviors, such as a sudden lane change or a drastic change in speed, of the front vehicle(s), and a wider road with more numbers of lanes provides an wider space to avoid a fierce collision under such circumstances. For the secondary CCs, hazards for a severe crash are received in two-ways: one is directly from the primary crash and the other is through (probably hazardous or abnormal) behaviors of the vehicles between them. In both cases, more lanes provide wider spaces to prepare and take action and can possibly reduce the severity level although a crash is still unavoidable.

A CC in a freeway tunnel had a 19.4% higher propensity to be severe than that occurring on open freeways (coefficient = 2.47, elasticity = 19.4). In its nature, a freeway tunnel possesses traffic hazards given its constraint driving space and tedious driving environment [48,49]. Poor lighting and fatigued driving in a tunnel are also often considered as potential hazards causing crashes, especially severe ones [50,51]. Based on the definition, a secondary CC happens very shortly after the primary crash occurs, meaning that there is extremely limited time for the following drivers to react to the primary crash. Hence, the poor driving environments and the fatigued-prone mental and physical conditions of the drivers in tunnels may result in a belated reaction than normal and thus cause severe CCs.

For the various weather conditions estimated in the two-level binary logistic regression model for CC severity, rainy (coefficient = 2.84) and cloudy (coefficient = 2.42) showed significant positive effects at the 95% confidence level or above. Compared with sunny days, a CC happening on a rainy day had a 52.5% higher possibility to be severe, and that happening on a cloudy day had a 66.6% higher possibility to be severe. These two types of extreme weathers display similar associations with crash severity in other contexts and can both incur poor visibility and hinder drivers’ perceptions and reactions to potential hazards or a primary crash, and thus causes more severe CCs [19,46,52,53]. Besides, pavements of freeways tend to be more slippery on rainy days [19,20,46], probably leading to a longer braking distance, and consequently result in a severe CC. It is worth noting that previous research found that drivers tended to drive more carefully under hazardous weathers such as on rainy days and thus probabilities for crash fatalities on rainy and cloudy days are lower especially during the day [54]. Based on the results in our study, although skillful drivers may get accustomed to extreme weathers promptly and drive carefully, the secondary CCs happen “within a blink” after the primary crash, and the objective elements (i.e., the existing hazards of extreme weathers) play a more crucial rule and cause more severe crash outcomes.

## 5. Conclusions

This study investigated the influential factors associated with the severity of crashes in a CC series. The severity of CCs occurring on the freeway network of Guizhou Province, China in 2018 were adopted in the modeling. Four model forms, including basic, RE, RP, and two-level binary logistic regressions were proposed to link the probability of a severe CC to the potential influential factors (i.e., environmental factors and crash-related attributes). To identify the specific influential factors affecting secondary CC severity, interactions between the dummy indicator for secondary CC and various factors (i.e., environmental factors and primary crash attributes) were created and incorporated in the models. The two-level logistic model was proven to outperform the other three model forms for modeling CC severity with our dataset, according to modeling performance metrics such as log-likelihood, McFadden Pseudo $R^{2}$ and the AIC value.

According to the coefficient estimates of the two-level logistic model, significant influential factors affecting CC severity were discussed. On the crash-level, the involvement of trucks in a secondary CC had a higher probability for it to be severe, and a primary CC with two vehicles involved had a significantly lower probability to incur a severe secondary CC than that with three or more vehicles. The facts that a secondary CC occur very shortly after the primary CC and that prompt reactions are crucial to its crash severity help to explain the crash-related results. On the road-segment level, environmental factors such as speed limit, traffic volume, tunnel, and rainy and cloudy weathers had significantly positive associations with CC crash severity. The number of lanes, on the contrary, had a negative effect on the severity of CC as a larger road space prepares the following vehicles for a possible escape from a severe hitting.

As discussed before, CCs contains multiple crashes can typically affects a large area of the road and paralyses the traffic system. Evacuations of the injured are normally difficult, especially if severe congestions are formed. Hence, precautions for lowering the crash severity of CCs are crucial. Based on the modeling results of this study, some policy suggestions can be made to reduce the severity levels of CCs. First, drivers should be reminded with hazardous situations where severe CC tend to occur. Dynamic warning signs should be placed in freeway tunnels and two-lane open roads with high speed limits (120 km/h in the case of China), especially in extreme weather such as on rainy and cloudy days. Similar warning systems are suggested to be inserted into mobile applications for navigation or the navigation systems in the vehicles to remind drivers of keeping a long-enough headway and alert of the potential hazards in the front. Besides, truck drivers are advised to experience trainings theoretically and practically with the potentiality for severe CCs to follow if they are involved in a primary crash. The truck drivers’ responsibility in maintaining a healthy driving environment on the road and preventing severe subsequent CCs from happening should be strengthened.

Based on local policies on crash data disclosure in Guizhou Province, only the data from 2018 was adopted in this study. Future works could benefit from applying multi-year data and study the temporal effects for CC severity [23]. Besides, the temporal threshold for the identification of CCs in this study is subject to minimum time interval (1 min) between two crash records in the database. Although 1 min is considered short enough as the behaviors and hazards induced by the primary crash needs time to spread, future studies are suggested to further split the temporal threshold into shorter periods and define the secondary CC in each period distinctively (i.e., CC occurring within 30 s, CC occurring 30 s to 1min, …), and compare the patterns in the severity of these subgroups of secondary CCs.

## Figures and Tables

**Table 1:** Descriptive statistics of dependent and independent variables.

Variable Name	Category/Explanation	Mean/Percentage	Standard Deviation
**Dependent Variable**			
Crash severity	Severe	33.1%	
	Non-severe	66.9%	
**Independent Variables**			
Environmental factors:			
Speed limit	km/h	116.1	11.7
Number of lanes		2.1	0.3
Traffic volume	10^6^	2.2	0.8
Weekend	Yes	30.5%	
	No ^†^	69.5%	
Weather	Rainy	38.7%	
	Cloudy	52.1%	
	Sunny ^†^	9.2%	
Location	Tunnel	21.4%	
	Bridge	2.4%	
	Others ^†^	76.2%	
Crash attributes:			
Secondary CC	Yes	61.2%	
	No ^†^	38.8%	
Truck involvement	Truck involved	19.0%	
	No truck involved ^†^	81.0%	
Trailer truck involvement	Yes	4.3%	
	No ^†^	95.7%	
Bus involvement	Yes	2.1%	
	No ^†^	97.9%	
Crash type	Rear-end	62.0%	
	Rollover	6.4%	
	Side-swipe	2.7%	
	Hitting objects ^†^	28.9%	
Number of vehicles involved	Single-vehicle	42.2%	
	Double-vehicle	34.0%	
	Multi-vehicle ^†^	23.8%	
Attributes of the primary crash in the CC series:			
Crash type of the primary crash	Rear-end	59.6%	
	Rollover	6.4%	
	Side-swipe	0.6%	
	Hitting objects ^†^	33.4%	
Number of vehicles involved in the primary crash	Single-vehicle	52.4%	
	Double-vehicle	30.2%	
	Multi-vehicle ^†^	17.4%	
Truck involvement in the primary crash	Yes	20.9%	
	No ^†^	79.1%	
Trailer truck involvement in the primary crash	Yes	6.1%	
	No ^†^	95.9%	
Bus involvement in the primary crash	Yes	2.4%	
	No ^†^	97.6%	

^†^ Reference group.

**Table 2:** Estimation results for the proposed four model forms.

	Basic binary Logistic Model			RE Logistic Model			RP Logistic Model			Two-Level Logistic Model		
Variables	Mean	SD	*p*-Value	Mean	SD	*p*-Value	Mean	SD	*p*-Value	Mean	SD	*p*-Value
Intercept	–4.41	2.99	0.139	–4.42	2.99	0.139	–4.01	4.37	0.359	–2.69	3.46	0.437
Environmental Factors												
Speed limit	0.40 **	0.01	0.007	0.40 **	0.01	0.007	0.06 **	0.02	0.016	0.04 **	0.02	0.035
Number of lanes	−2.91 **	1.04	0.005	−2.91 **	1.04	0.005	−4.90 **	1.99	0.014	−4.29 **	1.29	0.008
Traffic volume	0.72 **	0.20	<0.001	0.72 **	0.20	<0.001	1.14 **	0.35	0.001	1.41 **	0.40	0.004
Rainy	3.24 **	1.13	0.004	3.24 **	1.13	0.004	4.22 **	1.35	0.002	2.84 **	1.26	0.024
Cloudy	2.74 **	1.11	0.014	2.74 **	1.11	0.014	2.96 **	1.27	0.020	2.42 **	1.20	0.044
Tunnel	1.97 **	0.66	0.003	1.97 **	0.66	0.003	2.25 **	0.94	0.016	2.47 **	0.63	<0.001
Bridge	2.62 **	1.10	0.017	2.62 **	1.10	0.017	3.72 **	1.58	0.018			
Crash attributes												
Truck involvement	0.67 *	0.38	0.075	0.67 *	0.38	0.075	0.83 *	0.48	0.085			
Trailer truck involvement	1.99 **	0.73	0.006	1.99 **	0.73	0.006	3.06 **	1.14	0.007			
Interactions												
Single-vehicle primary crash × secondary CC	−0.11	0.34	0.748	−0.11	0.34	0.748	−0.61	0.50	0.222	0.09	0.55	0.875
Two-vehicle primary crash × secondary CC	−1.33 **	0.52	0.010	−1.33 **	0.52	0.010	−2.15 **	0.81	0.008	−1.80 **	0.87	0.012
Weekend × secondary CC	−1.01 **	0.43	0.017	−1.01 **	0.43	0.017	−1.45 **	0.64	0.023			
Tunnel × secondary CC	1.31 *	0.71	0.064	1.31 *	0.71	0.064	2.48 *	1.15	0.031			
Truck involvement × secondary CC										2.80 **	0.87	0.001
Random Effects (SD)												
Cloudy							2.75 **	1.06	0.009			
Double-vehicle primary crash × secondary CC										0.002	1.22	0.989
Truck involvement × secondary CC										4.24 × 10^−5^	0.89	0.999
$\sigma_{v}$ (random intercept)				0.23	1.13	0.984				1.00 **	0.50	0.045
ρ				0.13	――	――						

* means the estimated coefficient is significant at the 90% confidence level. ** means the estimated coefficient is significant at the 95% confidence level or above.

**Table 3:** Model comparison based on log-likelihood, McFadden Pseudo $R^{2}$, and AIC.

Model	Log-Likelihood at Convergence	McFadden Pseudo R^2^	AIC
Basic logistic model	−162.70	――	353.4
RE logistic model	−162.70	0.372	355.4
RP logistic model	−159.88	0.383	349.8
Two-level logistic model	−149.68	0.423	325.4

**Table 4:** Elasticity analyses results for the two-level binary logistic model.

Variables	Mean	SD	*p*-Value
Intercept	–1.21 **	0.02	<0.001
Speed limit	2.60 **	0.07	<0.001
Number of lanes	−5.11 **	0.17	<0.001
Traffic volume	1.64 **	0.06	<0.001
Rainy	52.5 **	4.00	<0.001
Cloudy	66.6 **	4.12	<0.001
Tunnel	19.4 **	2.28	<0.001

** means the estimated coefficient is significant at the 95% confidence level or above.
